# No evidence that mask-wearing in public places elicits risk compensation behavior during the COVID-19 pandemic

**DOI:** 10.1038/s41598-022-05270-3

**Published:** 2022-01-27

**Authors:** Lasse S. Liebst, Peter Ejbye-Ernst, Marijn de Bruin, Josephine Thomas, Marie R. Lindegaard

**Affiliations:** 1 Department of Sociology, University of Copenhagen, Copenhagen, Denmark; 2 NSCR: Netherlands Institute for the Study of Crime and Law Enforcement, Amsterdam, The Netherlands; 3 Department of Sociology, University of Amsterdam, Amsterdam, The Netherlands; 4 National Institute of Public Health and the Environment (RIVM), Bilthoven, The Netherlands; 5 Radboud University Medical Center, Radboud Institute of Health Sciences, Nijmegen, The Netherlands

**Keywords:** Epidemiology, Infectious diseases

## Abstract

Face masks have been widely employed as a personal protective measure during the COVID-19 pandemic. However, concerns remain that masks create a false sense of security that reduces adherence to other public health measures, including social distancing. This paper tested whether mask-wearing was negatively associated with social distancing compliance. In two studies, we combined video-observational records of public mask-wearing in two Dutch cities with a natural-experimental approach to evaluate the effect of an area-based mask mandate. We found no observational evidence of an association between mask-wearing and social distancing but found a positive link between crowding and social distancing violations. Our natural-experimental analysis showed that an area-based mask mandate did not significantly affect social distancing or crowding levels. Our results alleviate the concern that mask use reduces social distancing compliance or increases crowding levels. On the other hand, crowding reduction may be a viable strategy to mitigate social distancing violations.

## Introduction

During the COVID-19 pandemic, most countries recommend or mandate the use of face masks in public places. This measure aligns with a growing consensus that mask-wearing by members of the public is effective in mitigating coronavirus transmission^1,2^. However, there remain concerns that mask use may have unintended adverse behavioral effects, including that mask-wearing creates a false sense of security, which reduces compliance with other key mitigation measures^3,4^. Often this is attributed to a risk compensation mechanism that leads individuals to behave riskier in situations they perceive as safer^5^. Early in the pandemic, the World Health Organization expressed concerns along these lines^3^, which was also a focal point of the arguments for and against face masks in the Dutch national context of the current study^6^.

While these concerns may have played a role in delaying the adoption of personal protective measures during the COVID-19 pandemic^7^, the evidence supporting the risk compensation hypothesis remains controversial, fragile, and has been met with considerable scholarly criticism during the pandemic^8^. For example, the wearing of ski or bicycle helmets—often presented as a paradigmatic example of risk compensation—is not robustly linked with more risky practices^9,10^, and similarly, the evidence does not support that mask use adversely affects hand hygiene^7^. Nevertheless, mask use has been speculated to adversely affect social distancing directives^11^—for example, avoiding physical contacts and minimizing gatherings^12^. The assumption is that if individuals wearing masks feel protected, they may adhere less to social distancing directives, and likewise, other persons may feel that it is safe to have close encounters with a mask-wearer^13^.

Since the onset of the COVID-19 pandemic, several studies have offered conflicting evidence for risk compensation with respect to social distancing and related behaviors. Contrary to the risk compensation hypothesis, a number of field experiments found that people tend to keep a greater or a similar social distance to masked than unmasked persons^14–17^. For example, one study demonstrated that subjects kept a greater distance to a masked confederate queuing in front of a shop compared to an unmasked confederate. Additionally, it was reported that masked persons themselves did not keep a shorter distance from others and that mask mandates did not lead to less distancing compliance^15^. Only one field experimental study found conditional support of risk compensation, e.g., with men keeping less distance to masked confederates^18^. Comparatively, studies relying on other methods tend to offer more mixed evidence^19,20^. For example, one survey study found that mask-wearing was linked with greater concern about avoidance of others in public places^21^, while a quasi-experimental survey study found fragile evidence that mask use may occasionally elicit risk compensation by reducing the engagement in physical distancing^20^. Further, difference-in-differences studies relying on geo-tracked mobility data found that compulsory mask policies did not affect community mobility^22^ and, conversely, that such mandates lead to less stay-at-home compliance^4^.

Overall, the literature offers a somewhat mixed picture of the link between mask use and social distancing, although it is also noteworthy that field-experimental evidence relying on direct records of real-life mask use and social distancing behavior tend to agree in rejecting the hypothesis. Following this lead, the current study applies an alternative method to provide high-resolution data on real-life public behavior: video-assisted naturalistic observation^23^. Specifically, across two studies—one observational and one that includes a natural experiment in two major Dutch cities—we used video footage to examine mask use and distancing behavior in public outdoor settings during the COVID-19 pandemic. We tested the individual-level expectation of the risk compensation theory that mask-wearers keep less distance, as well as the parallel expectation at the aggregated level that an area-based mask mandate would make public places more crowded. We note that the study is exploratory in nature (in that an analysis plan was not pre-registered), but we stress that we strived to minimize the number of researcher degrees of freedom in the analysis and were committed to reporting our results irrespective of null findings^24^.

## Study 1

### Methods

Data were a sample of video observed individuals recorded by municipality-operated public security cameras in the Netherlands (data and materials are available at osf.io/j7guw). We obtained access to more than 60,000 h of footage across 63 cameras located in Amsterdam. Given that manual video coding is very labor-intensive, we selected 60 h of recording from this wider sample^25^, which conformed to the following inclusion criteria: The clips were recorded by a single camera to minimize between-context heterogeneity. This camera should have a high recording quality (e.g., brightness, resolution), allow for continuous observation of pedestrians from a long distance, and be located in an outdoor pedestrianized street typical for Amsterdam (no side roads, traffic lights, crossings, and pedestrians only, see osf.io/j7guw for street drawings). Further, data should be recorded during the first wave of the pandemic (i.e., May 21, 24, 28, 30, and June 4, 2020), the day hours (to ensure the recording brightness), and cover both week- and weekend days (i.e., three Thursdays, one Saturday, one Sunday) and their varying behavioral routines^26^.

#### Coding procedure

Two trained research assistants coded data following a codebook developed for the study. The interrater reliability of the codebook was evaluated by independently double coding 44 individuals, nested across 25 video-recorded situations (with disagreements resolved among the coders). All included variables had a Gwet’s^27^ AC_1_/AC_2_ score larger than 0.8, indicating excellent agreement (each score is noted in the below Measures section).

The coding began by randomly selecting 51 30-min segments across the 60 h of footage included. If possible, for each segment, we then observed seven persons with a mask and—to construct a relatively balanced sample—seven persons without a mask entering and leaving the scene (for the large majority of cases, the observation lasted as long as they were in camera view). In total, we sampled 383 persons (176 with and 207 without a mask), observed on average for 25 s (*SD* = 7.4). This satisfied an a priori power analysis suggesting that 339 cases would detect a small effect (f^2^ = 0.05), with a power of 90% and a conservative alpha of 0.005^28^. We did not design the study to detect effects below this effect size threshold, given that this would increase the required *N* significantly and we had limited recourses available for coding^29^. Note that we coded beyond the required number of observations to have a buffer for missing data.

#### Measures

The dependent variable was captured as a binary variable distinguishing between whether or not the observed individual was within a 1.5 m radius of a stranger (AC_1_ = 0.92), i.e., the official Dutch meter-threshold for social distancing. Whether the other person is a stranger or affiliated was inferred from whether they arrived at the scene together and walked in each other’s company^30^. To assess the coding of interpersonal distance, we utilized the exact dimensions of street tiles as a “ruler.” Note that we also, as an alternative “high-risk” version of the dependent variable, measured social distancing with a 0.5 m cut-point (AC_1_ = 0.89) (i.e., proximity within other’s intimate space^31^).

The independent variable was a binary measure, distinguishing whether the person wore a face mask or not (AC_1_ = 1.0). Face masks included respirators (e.g., N95), surgical masks, cloth masks, and excluded persons wearing face shields and improvised face coverings (e.g., bandanas, scarves). We also excluded persons wearing masks covering neither the nose nor the mouth (e.g., hanging under the chin) or who changed the mask’s placement (i.e., between facial areas or putting it on/off). Finally, we included some control variables in the observational analysis: A visual assessment of the person’s age (AC_2_ = 0.90) and gender (AC_1_ = 0.96), given evidence indicating that these individual factors may influence social distancing compliance^18,32^. Further, we accounted for crowding—captured as a count of the number of persons moving through each segment (AC_2_ = 1.0)—because of evidence demonstrating an association between crowding levels and social distancing violations^33^.

#### Estimation

All analyses were executed with Stata 16^34^. Regression models were estimated with linear probability models^35^, specified with cluster-robust standard errors where possible (i.e., individuals nested in observation segments). For regression results yielding non-significant *p*-values (i.e., *p* < 0.005)^28^, we also reported Bayes factor (BF) to evaluate whether data either supported the null-hypothesis or indicated data insensitivity in distinguishing the null and alternative hypotheses (i.e., a distinction that cannot the established from a non-significant *p*-value)^36^. The Bayes factors were approximated from Bayesian Information Criterion (BIC) values^37,38^, assuming a vague unit-information prior (i.e., a prior that carries as much information as a single observation^39^).

#### Ethics and approvals

This study was approved by relevant authorities. The study was validated by The Ethics Committee for Legal and Criminological Research at the Vrije University Amsterdam. The Amsterdam Police Department provided data access with the approval of the Netherlands Public Prosecution Service. The Danish Data Protection Agency further approved the study’s compliance with the General Data Protection Regulation (GDPR) of the European Union. Note that we did not obtain informed consent from the video-captured persons, given that this was neither practically feasible nor required under the GDPR nor an ethical requisite (see Sect. 8.03 of The American Psychological Association’s Ethical Principles^40^).

### Results

**Figure 1:**
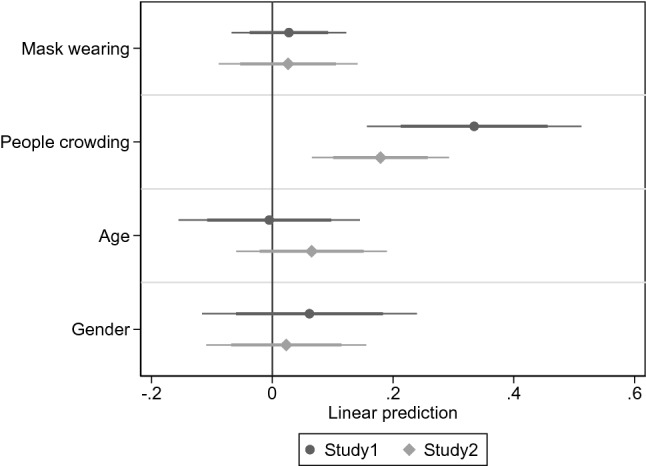
Regression analyses of observational data of social distancing violations in Study 1 and Study 2. Note. Linear probability model estimates, with 95% and 99.5% confidence intervals (two-tailed). All models controlled for the time duration of each observation. The continuous age and crowding items were standardized to make them comparable to binary predictors^59^.

Across the average observation window of 25 s, more than half (*M* = 0.54, *SD* = 0.50) of the observed individuals were involved in at least one 1.5 m distancing violation—a noticeably high incident when considering the short observation window. Around one out of ten (*M* = 0.12, *SD* = 0.45) was involved in violations if social distancing was alternatively defined with a 0.5 m cut point. Figure 1 summarizes the regression results. We see that mask-wearing was not associated with 1.5 m distancing violations (β = 0.03, CI 95% [-0.04, 0.09], *p* = 0.398), with a Bayes factor suggesting that data were 16.5 times more likely to occur under the null than the alternative hypothesis—that is, strong evidence for the absence of an association, evaluated with the thresholds:^38^ weak < 3, substantial ~ 3, strong ~ 10, very strong ~ 30. Further, crowding was a significant positive predictor of social distancing violations (β = 0.33, CI 95% [0.21, 0.46], *p* < 0.001). The full impact of crowding was linked with a 33-percentage increased likelihood of distancing violations, a substantial effect size (note that crowding estimated with a nonlinear quadratic form was non-significant, but see further^41^). The controls for age (β = − 0.01, CI 95% [− 0.11, 0.10], *p* = 0.922, BF_01_ = 13.4) and gender (β = 0.06, CI 95% [− 0.06, 0.18], *p* = 0.314, BF_01_ = 5.5) were both non-significant.

## Study 2

### Methods

We designed study 2 as a replication of study 1 and, as such, applied the same overall coding strategy and measures as Study 1. However, Study 2 also utilized a natural-experimental situation^42^, with the municipalities of Amsterdam and Rotterdam implementing an area-based mask mandate. At the time, the Dutch government was against a nationwide mask mandate. However, city mayors were given the permission to take additional measures, and the cities of Amsterdam and Rotterdam took this opportunity to evaluate the social distancing effects of a mask mandate in crowded urban settings. This step was motivated by increasing infection numbers in both cities^43^.

The mask mandate was implemented in eight particularly crowded streets (i.e., tourist and shopping areas). Because the municipalities wanted an experimental evaluation, they did not implement the mandate in all eligible areas. Therefore, we selected control areas from a wider list of areas considered for treatment but eventually remained untreated. In sum, we selected three treatment areas and three comparable control areas, which had the best-quality public security cameras installed. Practically, the mask mandate was announced by onsite signs, municipal workers informing visitors and handing out masks during the first weeks, and police reprimanding or fining non-compliers for 1 day during the third week. Further, the mandate was heavily debated on national media.

We collected around 500 h of recordings, recorded from the end of July throughout August 2020. The raw footage covered 13 days (Wednesdays, Saturdays, one Sunday), with four days of pre-intervention baseline measures (July 22, 25, 29, and August 1), and nine post-intervention days (August 5, 8, 12, 15, 19, 22, 23, 26, 29). From this sample, we randomly selected 78 30-min segments, across which a team of twelve trained research assistants observed 423 persons (167 with and 256 without a mask) entering and leaving the scene, with an average person observation time of 23 s (*SD* = 17.8). Finally, at randomly selected time points across the treatment and control areas, we further took 358 records of crowding and 342 records of the proportion of people wearing a mask. Additionally, we conducted onsite observations in treatment areas to document how the mandate was implemented in practice (e.g., were the onsite signs announcing the mask mandate clearly visible)^44^.

In coding the data, we relied on the same codebook interrater-reliability tested in Study 1, assuming that the high scores reached in Study 1 suggested that the measures would be similarly applicable in the comparable Study 2 context. This decision was informed by the experience of training the coder team, with excises of independent double coding suggesting that the codebook was transferable to the new study context. However, it should be mentioned that the study contexts were not identical. Not all areas in Study 2 had street tiles—used as a “ruler” to assess social distancing in Study 1—and in these areas, the coders thus relied on alternative reference objects measured onsite. Finally, we note that the 1.5-m distancing measure has since been interrater reliability tested twice, both in areas without and with different types of tiles layers; in both evaluations, we reached excellent scores replicating those found in Study 1 (i.e., AC_1_ = 0.97, AC_1_ = 0.98^45^).

### Results

Across the average observation time of 23 s, around four out of five persons were engaged in 1.5 m distancing violations (*M* = 0.72, *SD* = 0.45) and approximately one in three (*M* = 0.30, *SD* = 0.46) passed someone with less than a 0.5 m radius. Both the 1.5 m (χ^2^ (1, *n* = 803) = 29.1, *p* < 0.001) and the 0.5 m (χ^2^ (1, *n* = 800) = 38.4, *p* < 0.001) violations were significantly more common in Study 2 compared to Study 1. There were no significant between-study differences with respect to age (*t*(381) = − 1.5, *p* = 0.148), gender (χ^2^ (1, *n* = 803) = 2.8, *p* < 0.094), and crowding (*z* = − 1.31, *p* = 0.192).

Figure 1 shows that the key regression results of Study 2 were similar to Study 1: Mask use was again not associated with social distancing (β = 0.03, CI 95% [− 0.05, 0.10], *p* = 0.511), with a Bayes factor offering strong evidence for the absence of this association (BF_01_ = 17.0). Also, people crowding was positively associated with social distancing violations (β = 0.18, CI 95% [0.10, 0.26], *p* < 0.001). These results remained unchanged after controlling for whether the persons were in an area where mask-wearing was voluntary or mandatory. To minimize the risk that Study 1 and Study 2 were underpowered to identify a potential minute effect of masks on distancing when estimated separately, we analyzed a dataset pooled from the two studies^46^. This analysis confirmed the non-significant result of masks (β = 0.01, CI 95% [− 0.04, 0.07], *p* = 0.584, BF_01_ = 25.5).

Next, the natural-experimental data was analyzed with difference-in-differences regression models^47^ appropriate for the outcome type (note that nonlinear interaction effects were tested as second differences^48^; for further details, see osf.io/j7guw). A manipulation check found that the area-based mask mandate increased the proportion of mask-wearing by more than 30 percentage points (second difference = 0.32, *p* < 0.001), suggesting a relatively successful implementation of the treatment. The predicted probability of mask-wearers in the pre-intervention treatment condition was 3% and 39% in the post-intervention condition. The non-complete adherence to the mandate corresponded with our field observations conducted in the treatment areas, suggesting some uncertainty about where it was obliged to wear a mask (e.g., the signs announcing that you entered a mandatory mask zone could be overlooked; plausibly, this may have impacted one of the included cameras, which was placed at the fringe of a zone).

We found that the mask mandate did not affect the individual-level likelihood of social distancing encounters (β = 0.036, CI 95% [− 022, 029], *p* = 0.781, BF_01_ = 18.8), and this result remained non-significant after controlling for crowding. Further, the mask mandate treatment was not associated with the level of people crowding (second difference = − 5.77, *p* = 0.126, BF_01_ = 3.3). We highlight that this result, to some extent, hinged on how the models were specified. Specifically, when specified without cluster-robust standard errors—which, however, is recommended^49^—the model yielded a marginally significant negative difference-in-differences estimate (i.e., direct counterevidence for the risk compensation hypothesis).

## Discussion

It would be a cause for concern if face masks reduced the adherence to social distancing directives, as predicted by the risk compensation hypothesis. The current study helps alleviate that concern, with internally replicated observational evidence for the absence of a mask-distancing association and natural-experimental data showing that a mask mandate was not associated with social distancing and crowding levels.

Although the literature on risk compensation around mask use is sparse and of somewhat mixed quality, studies relying on field experiments—a method particularly well-suited to evaluate behavioral influences in naturally occurring settings^50^—tend to reject the risk compensations hypothesis^14–17^. The current observational and experimental studies—examining both voluntary and mandatory mask settings—also rejected this hypothesis. As such, our findings add valuable information to the literature suggesting that face mask-wearing is not likely to lead to a reduction in social distancing compliance or an increase in crowding levels due to a false sense of security. With accumulating evidence that mask-wearing is efficient in reducing the spread of coronavirus^51^, the current results add to the view that this beneficial mask effect is unlikely to be “canceled out” by an adverse risk compensation effect of masks-wearing in public settings.

As an alternative to a risk-compensation explanation of social distancing, the current results suggest—in line with prior research^32,33^—that social distancing violations are chiefly predicted by crowding. That is, when citizens move through crowded public places, it is more challenging to keep the desired 1.5 m distance to other people. This result indicates that the literature has been overly focused on individual-level predictors of social distancing violations (e.g., risk compensation, age, norms) at the expense of analyzing the “situational opportunity” for violations afforded by people crowding^26^. A practical implication of this argument is that COVID-19 interventions towards social distancing violations may find utility in crowd control^52^ and in targeting infrastructural surroundings that shape public crowding^53^. In doing so, however, it should be kept in mind that the risk of coronavirus transmission is lower in outdoor than indoor settings^54^. As such, crowd control interventions may be most relevant for enclosed semi-public places (e.g., train stations, shopping malls) where some prior research also finds that crowding decreases interpersonal distance^55^.

A limitation of the current study is that we do not distinguish whether it is the potential mask-wearer or their counterparts who have the primary role in stepping over the 1.5 m distancing threshold. This issue arises because both parties were often moving, i.e., in contrast to field experimental studies exploiting how the test subjects moved with a certain distance towards a masked or unmasked confederate standing in a queue^15^. Another limitation is the between-subject design—especially in the non-experimental Study 1—which cannot separate the effect of mask-wearing from the potential selection-effect that mask-wearers are more (or less) conscientious persons and willing to distance. For example, the willingness to distance varies for some persons across the pandemic^56^, as plausibly indicated by the circumstance that the compliance level was higher during the first wave (Study 1) than during the second wave (Study 2).

Further, we acknowledge the *quasi*-experimental nature of Study 2, with a non-random assignment of the area-based treatment and, thus, an inflated risk that our results are affected by unaccounted confounders. Note, however, that the analysis did account for a potential key confounder—i.e., crowding—that we identified in our observational analyses. We also acknowledge that our experimental approach is constrained by a short follow-up period of four weeks and that the reported null findings may be due to an insufficiently powerful treatment, as indicated by the only partially successful manipulation check. Finally, it should be mentioned that the null results of the difference-in-differences analyses may be due to us underestimating how large a sample should be to identify interaction effects (i.e., around four times larger than the main effects^57^). As such, these tests were de facto powered to identify ‘medium’-sized interaction effects (rather than a ‘small’-sized main effects as planned). This, in turn, may have inflated the false-negative error risk of the difference-in-differences analyses.

In conclusion, the current studies provided observational and natural-experimental evidence that mask-wearing does not reduce social distancing or increase crowding, neither under voluntary nor mandatory conditions.

## Data Availability

Data and materials are available at osf.io/j7guw.

## Additional Information

Note that the wider data sample—from which the videos included in Study 1 and 2 originated—were collected as part of a larger research project, within which several papers draw on overlapping or other videos to address different study objectives or analyze them using other methods^33,41,45,58^.
